# Supplementary material for: Risk of Schizophrenia and Bipolar Disorder in Patients With Multiple Sclerosis: Record-Linkage Studies
Source: Front Psychiatry. 2020 Jul 16;11:662. doi: 10.3389/fpsyt.2020.00662 (PMC7378813; doi:10.3389/fpsyt.2020.00662)
Supplement: Supplementary Table 1 — Associations between multiple sclerosis (MS) and schizophrenia (SZ) or bipolar disorder (BP) in Females, English national Hospital Episode Statistics, 1999–2016. [file DataSheet_1.docx]

**Supplementary Table 1: Associations between multiple sclerosis (MS) and schizophrenia (SZ) or bipolar disorder (BP) in Females, English national Hospital Episode Statistics, 1999-2016**

|  |  |  |  | Exposure and outcome taken from anywhere on the record | | | | |  | Exposure and outcome as primary diagnosis only | | | | |
| --- | --- | --- | --- | --- | --- | --- | --- | --- | --- | --- | --- | --- | --- | --- |
| Exposure | Outcome | Follow-up interval | | Total | Observed | HR | 95%CI | p value |  | Total | Observed | HR | 95%CI | p value |
| BP | MS | All |  | 125790 | 287 | 1.65 | (1.47-1.86) | 0.00000 |  | 55440 | 70 | 1.29 | (1.01-1.63) | 0.03776 |
| MS | BP |  |  | 89282 | 435 | 1.22 | (1.11-1.35) | 0.00003 |  | 44983 | 65 | 0.97 | (0.76-1.24) | 0.80726 |
| SZ | MS |  |  | 174967 | 317 | 1.33 | (1.19-1.49) | 0.00000 |  | 98153 | 80 | 0.97 | (0.77-1.21) | 0.76324 |
| MS | SZ |  |  | 89282 | 512 | 1.62 | (1.49-1.77) | 0.00000 |  | 44983 | 98 | 1.18 | (0.97-1.45) | 0.09783 |
|  |  |  |  |  |  |  |  |  |  |  |  |  |  |  |
| BP | MS | <1yr |  | 125790 | 47 | 2.10 | (1.56-2.81) | 0.00000 |  | 55440 | 7 | 1.22 | (0.58-2.59) | 0.59643 |
| MS | BP |  |  | 89282 | 71 | 1.81 | (1.43-2.29) | 0.00000 |  | 44983 | 14 | 1.54 | (0.9-2.62) | 0.11195 |
| SZ | MS |  |  | 174967 | 54 | 1.77 | (1.35-2.33) | 0.00004 |  | 98153 | 13 | 1.49 | (0.86-2.6) | 0.15633 |
| MS | SZ |  |  | 89282 | 72 | 1.71 | (1.35-2.16) | 0.00001 |  | 44983 | 18 | 1.56 | (0.98-2.49) | 0.06331 |
|  |  |  |  |  |  |  |  |  |  |  |  |  |  |  |
| BP | MS | 1yr+ |  | 109753 | 240 | 1.59 | (1.4-1.81) | 0.00000 |  | 52207 | 63 | 1.29 | (1.01-1.66) | 0.04399 |
| MS | BP |  |  | 79940 | 364 | 1.15 | (1.04-1.28) | 0.00856 |  | 41410 | 51 | 0.88 | (0.67-1.16) | 0.36519 |
| SZ | MS |  |  | 150661 | 263 | 1.26 | (1.12-1.43) | 0.00020 |  | 91135 | 67 | 0.90 | (0.71-1.15) | 0.41382 |
| MS | SZ |  |  | 79941 | 440 | 1.61 | (1.46-1.77) | 0.00000 |  | 41406 | 80 | 1.12 | (0.9-1.4) | 0.30093 |

**Supplementary Table 2: Associations between multiple sclerosis (MS) and schizophrenia (SZ) or bipolar disorder (BP) in Males, English national Hospital Episode Statistics, 1999-2016**

|  |  |  |  | Exposure and outcome taken from anywhere on the record | | | | |  | Exposure and outcome as primary diagnosis only | | | | |
| --- | --- | --- | --- | --- | --- | --- | --- | --- | --- | --- | --- | --- | --- | --- |
| Exposure | Outcome | Follow-up interval | | Total | Observed | HR | 95%CI | p value |  | Total | Observed | HR | 95%CI | p value |
| BP | MS | All |  | 77802 | 112 | 1.98 | (1.64-2.39) | 0.00000 |  | 38932 | 33 | 1.39 | (0.98-1.97) | 0.06112 |
| MS | BP |  |  | 38912 | 96 | 0.85 | (0.69-1.04) | 0.11247 |  | 20527 | 12 | 0.49 | (0.28-0.86) | 0.01348 |
| SZ | MS |  |  | 209221 | 182 | 1.14 | (0.98-1.33) | 0.08309 |  | 129654 | 50 | 0.60 | (0.46-0.8) | 0.00053 |
| MS | SZ |  |  | 38912 | 207 | 1.11 | (0.97-1.27) | 0.13741 |  | 20527 | 44 | 0.79 | (0.58-1.06) | 0.11344 |
|  |  |  |  |  |  |  |  |  |  |  |  |  |  |  |
| BP | MS | <1yr |  | 77802 | 22 | 3.21 | (2.09-4.95) | 0.00000 |  | 38932 | 7 | 2.99 | (1.4-6.41) | 0.00474 |
| MS | BP |  |  | 38912 | 20 | 1.51 | (0.97-2.35) | 0.06768 |  | 20527 | 2 | 0.59 | (0.15-2.38) | 0.46136 |
| SZ | MS |  |  | 209221 | 41 | 2.41 | (1.74-3.34) | 0.00000 |  | 129654 | 6 | 0.77 | (0.34-1.75) | 0.53260 |
| MS | SZ |  |  | 38912 | 45 | 1.65 | (1.23-2.22) | 0.00085 |  | 20527 | 15 | 1.80 | (1.08-3.01) | 0.02387 |
|  |  |  |  |  |  |  |  |  |  |  |  |  |  |  |
| BP | MS | 1yr+ |  | 67880 | 90 | 1.81 | (1.47-2.24) | 0.00000 |  | 36369 | 26 | 1.22 | (0.82-1.79) | 0.32423 |
| MS | BP |  |  | 34210 | 76 | 0.76 | (0.61-0.95) | 0.01783 |  | 18745 | 10 | 0.47 | (0.25-0.88) | 0.01804 |
| SZ | MS |  |  | 185311 | 141 | 0.99 | (0.83-1.17) | 0.88729 |  | 121785 | 44 | 0.59 | (0.43-0.79) | 0.00057 |
| MS | SZ |  |  | 34194 | 162 | 1.02 | (0.87-1.19) | 0.84353 |  | 18734 | 29 | 0.61 | (0.42-0.88) | 0.00774 |
